# Trends of Minimally Invasive Hysterectomy: Five Years of Experience

**DOI:** 10.1155/ogi/2366445

**Published:** 2025-06-12

**Authors:** Abdulrahman Khinkar, Afaf Felemban, Rahaf AlSomali, Norah AlSunayen, Ahmed Felemban, Joud Makki, Ghadeer Aljahdali

**Affiliations:** ^1^Department of Obstetrics and Gynecology, Women's Health Specialized Hospital, Riyadh, Saudi Arabia; ^2^College of Medicine, King Saud bin Abdulaziz University for Health Sciences, Riyadh, Saudi Arabia

**Keywords:** complications, laparoscopy, minimally invasive hysterectomy, retrospective, trend

## Abstract

**Objectives:** This study aims to investigate the five-year trend of minimally invasive hysterectomy and identify factors associated with increased intraoperative and postoperative complications within the last five years (2017–2021).

**Methods:** A retrospective chart review was performed between March 30th and April 10th, 2022, which included women who underwent total laparoscopic hysterectomy (TLH), laparoscopic subtotal hysterectomy (LSH), laparoscopic-assisted vaginal hysterectomy (LAVH), and robotic-assisted hysterectomy for benign gynecologic conditions, during the period January 2017 to December 2021.

**Results:** There was an increase in the rate of performing minimally invasive hysterectomy procedures from 35 cases (12.5%) in 2017 to 85 cases (30.5%) in 2021. In addition, abnormal uterine bleeding was the most reported indication for minimally invasive hysterectomy (*n* = 84; 30.1%). Estimated blood loss was a significant predictor for both intraoperative complications (*p* < 0.001) and postoperative complications (*p*=0.004).

**Conclusion:** Within the last five years, there has been an increasing trend of minimally invasive hysterectomy procedures at the OB-GYN department in KAMC, Saudi Arabia. And estimated blood loss is a significant predictor of intraoperative and postoperative complications of minimally invasive hysterectomy.

## 1. Introduction

Among the various surgical procedures for numerous gynecological conditions, hysterectomy is the most common, with the procedure being performed more than 600,000 times a year in the United States of America [1]. There is considerable variation in hysterectomy indications, and most of the indications are for benign gynecological diseases, including but not limited to uterine leiomyoma, abnormal uterine bleeding (AUB), pelvic organ prolapses (POP), pelvic pain or infection, and malignant and premalignant disease [2].

Surgical approaches to hysterectomy involve laparotomy, laparoscopy, as well as vaginal techniques. In the 19th century, it was first performed by vaginal or abdominal incision [3]. With the advent of modern technology, the first laparoscopic hysterectomy was performed in 1989 [4]. There are several types of minimally invasive hysterectomy, including total laparoscopic hysterectomy (TLH), laparoscopic subtotal hysterectomy (LSH), laparoscopic-assisted vaginal hysterectomy (LAVH), and robotic-assisted hysterectomy (RH) [5].

Several studies have examined the complication rate for laparoscopic hysterectomy for benign diseases and have reported it in a range of 5–14% [6, 7]. Based on extensive data from a systematic review [2], the estimates of specific complications are as follows: conversion to laparotomy (3.9%), urinary tract injury (1.2%–2.6%), vaginal cuff dehiscence (0.64%–1.1%), and bowel injury (0.34%–0.45%). Another study conducted in Denmark has shown that the complication rate is significantly higher in governmental hospitals where residents and fellows are being trained [8].

Over time, the trend toward minimally invasive gynecologic surgery has considerably expanded and developed [1], and the attitude toward abdominal hysterectomy has gradually shifted away from minimally invasive techniques [9]. The advantages of minimally invasive gynecologic surgery included shorter hospital stays, better postoperative pain, patient aesthetics, and a low complication rate, which made this technique more favorable compared to conventional laparotomy [10, 11].

In the United States, according to data from the nationwide inpatient sample (NIS), most hysterectomies were performed abdominally, as it has been reported that over 500 thousand hysterectomies were conducted for benign lesions in 2009, 64% abdominally, and only 14% laparoscopically [12]. In addition, a 5.8% reduction in performing minimally invasive hysterectomy in Florida has been reported [9].

Although extensive research has been carried out on the trend, complications, and indications of minimally invasive hysterectomy, data regarding that have not been extensively reported in Saudi Arabia. Therefore, this present study aims to investigate the 5-year trend of minimally invasive hysterectomy and identify factors associated with increased risk of intraoperative and postoperative complications.

## 2. Methods

This is a retrospective cohort study conducted at the King Abdullah Medical Center (KAMC) in Riyadh. The KAMC is regarded as one of the most comprehensive health care medical cities in Saudi Arabia, with a capacity of 1973 beds. The study was ethically approved by the Institutional Review Board of the King Abdullah International Medical Center (KAIMRC). The study registry number and the IRB number are as follows: H-01-R-005 and NRC22R/134/03.

The study included and examined the medical records of all women who underwent TLH, LSH, LAVH, and RH for benign gynecologic conditions at King Abdullah Medical City (KAMC) from January 2017 to December 2021. Patients who have performed hysterectomy for malignant indications or emergency procedures and those with incomplete medical records were excluded. Data were collected from KAMC's electronic medical record system called BESTCare.

Women were grouped based on the subtype of minimally invasive hysterectomy, and extracted data were collected through a datasheet that included demographics (age, weight, height, and body mass index “BMI”), clinical data (chronic medical disease: diabetes mellitus, hypertension, thyroid disease, and others, previous laparotomy, parity), type of laparoscopic hysterectomy, duration of the procedure, estimated blood loss, type of anesthesia, preoperative prophylactic antibiotic, use of prophylactic anticoagulant, indications, and intraoperative and postoperative complications of minimally invasive hysterectomy occurring within 30 days of surgery.

Febrile morbidity will be defined as more than or equal to 38 C requiring antibiotics, and operation time is defined as the time of skin surgical incision to the time of surgical skin closure.

All of procedures were conducted using standardized protocols by certified consultants (who were board-certified OB-GYN specialists with a minimum of five years of surgical experience, licensed by the Saudi Commission for Health Specialties (SCFHS)), including gynecologists and urogynecologists with varying levels of competence in which surgeons were categorized into three levels: junior (0–5 years of experience), mid-level (6–10 years), and senior (> 10 years). This stratification was used during complication analysis.

### 2.1. Data Analysis

The Statistical Package for Social Sciences (SPSS) (v. 25, IBM Corp. Chicago, IL, USA) was used for data analysis. Descriptive statistics were used to analyze the patients' baseline characteristics and the procedure-related data. In addition, binary logistic regression was used to investigate predictors of intraoperative and postoperative complications of minimally invasive hysterectomy. A significance level (*p* value ≤ 0.05) was used as a statistical significance threshold in this study.

## 3. Results

Table 1 represents the demographic and baseline characteristics of the study cohort (*n* = 279). The mean ± standard deviation (SD) of the age of enrolled patients was 53.3 ± 8.8, whereas their mean ± SD BMI was 33.7 ± 6.6. Moreover, a parity of more than six was observed among almost half of the patients (*n* = 134; 48.1%). About two-thirds (*n* = 180; 64.5%) had no previous laparotomy. History of chronic medical conditions was reported by 219 patients (78.5%), mainly diabetes mellitus (*n* = 99; 35.5%) and hypertension (*n* = 95; 34.1%).

**Figure 1:**
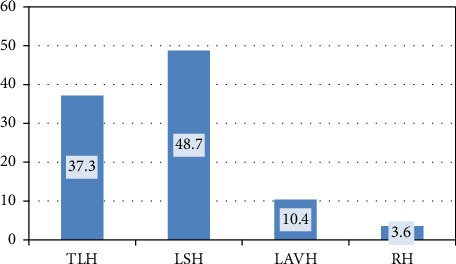
Type of minimally invasive hypersecretory. Total laparoscopic hysterectomy (TLH), laparoscopic subtotal hysterectomy (LSH), laparoscopic-assisted vaginal hysterectomy (LAVH), and robotic-assisted hysterectomy (RH).

With respect to the specific type of hysterectomy procedures, LSH ranked first (*n* = 136; 48.7%), followed by TLH (*n* = 104; 37.3%).  Figure 1.

Table 2 provides an overview of the minimally invasive hysterectomy procedure in the last five years (2017–2021). The results showed that 30.5% (*n* = 85) of the minimally invasive hysterectomy was performed in 2021, whereas in 2017, only 12.5% (*n* = 35) of the minimally invasive hysterectomy was performed. General anesthesia was adopted in the majority of minimally invasive hysterectomy procedures performed between 2017 and 2021 (*n* = 275; 98.5%). Preoperative prophylactic antibiotics were reported in 98.9% (*n* = 276) of procedures, whereas prophylactic anticoagulants were used in 273 cases (97.8%). The most frequently reported indications for minimally invasive hysterectomy were AUB (*n* = 84; 30.1%), POP (n-58; 20.8), postmenopausal bleeding (*n* = 55; 19.7%), and uterine leiomyoma (*n* = 55; 19.7%). Intraoperative complications were reported in 20 cases (7.2%), whereas postoperative complications were observed in 19 cases (6.9%). The duration of the procedure ranged between 1 and 7 h, with an arithmetic mean ± SD of 2.9 ± 1.0 h. Estimated blood loss ranged between 70 and 900 mL (263.0 ± 173.1), and postoperative hospital stays ranged between one and 25 days (2.2 ± 1.8).

**Figure 2:**
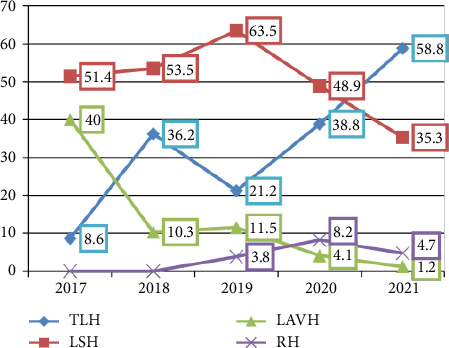
Trends of minimally invasive hysterectomy procedures over five years (2017–2021). Total laparoscopic hysterectomy (TLH), laparoscopic subtotal hysterectomy (LSH), laparoscopic-assisted vaginal hysterectomy (LAVH), and robotic-assisted hysterectomy (RH).

Following the five-year trend of the different types of minimally invasive hysterectomy, it was revealed that TLH increased significantly from three cases (8.6%) in 2017 to 50 cases (58.8%) in 2021. In contrast, LSH increased from 18 cases (51.4%) in 2017 to 31 cases (53.5%) in 2018 and 33 cases (63.5%) in 2019 and then declined to 30 cases (35.3%) in 2021. In addition, it was found that LAVH declined from 14 cases (40.0%) in 2017 to one case (1.2%) in 2021. Finally, RH was not performed in any case in 2017 and 2018 and in 4 cases ((8.2%) and (4.7%)) in 2020 and 2021, respectively.  Figure 2.

Table 3 revealed that the only factor that significantly predicted the occurrence of intraoperative complications was estimated blood loss (adjusted odds ratio = 1.005; 95% confidence interval: 1.001–1.01), *p* < 0.001. Also, the only factor that significantly predicted the occurrence of postoperative complications was estimated blood loss (adjusted odds ratio = 1.003; 95% confidence interval: 1.001–1.006; *p*=0.004; Table 4).

## 4. Discussion

This study evaluated the trend toward minimally invasive hysterectomy and whether there is a change in practice, complication rate, and indications. Our results suggest that the trend of minimally invasive hysterectomy has generally increased over five years (2017–2021), with LSH being the most significantly frequent procedure. This finding reflects the surgical practice transformation from open hysterectomy to minimally invasive hysterectomy and the distinguished increase in the adoption of minimally invasive hysterectomy over the traditionally open techniques demonstrated in previous studies [13, 14]. Additionally, most of the minimally invasive hysterectomies were performed in 2021, and moreover, the majority of minimally invasive hysterectomies were conducted in 2021, which can be ascribed to the recent arrival and hiring of skilled and certified gynecological surgeons specializing in minimally invasive procedures. This initiative provided a valuable chance for multiple OB/GYN consultants with an interest in minimally invasive gynecological surgery to enhance their skills in laparoscopic procedures through additional training.

Of the total 279 patients who underwent a minimally invasive hysterectomy, the mean age and BMI were higher than what has been demonstrated in previous studies [8, 15, 16]. The most common indications were AUB followed by postmenopausal bleeding, which contradicts what has been demonstrated in previous studies where the most common indication was uterine leiomyoma [17, 18]. The age group of most patients can explain such findings in this present study, as the majority are in menopausal transition or reached menopause.

Surgical complications were divided into two main categories, intraoperative and postoperative complications; out of the 20 patients who sustained intraoperative complications, 0.7% percent had bowel injury, 0.7% had a ureteric injury, and 2.5% of the laparoscopic procedure were converted to open laparotomy; these rates are comparable and similar to the currently existing studies [15, 19–21]. Therefore, this study demonstrated that bowel and ureteric injury rates are globally accepted for such complications. Regarding postoperative complications, out of 279 patients, 0.4% had a bowel obstruction; this result reflects those of Muffly et al., who also found that the risk of bowel obstruction was 0.53%, which is comparable to the present study [22].

Concerning febrile morbidity, 3 patients (1.1%) developed a fever that required postoperative antibiotics. This finding is similar to previous studies, where the rate ranges between 0% and 15% [23–25].

Generally, with a minimally invasive procedure, the estimated blood loss, operation time, and postoperative hospital stay are expected to be lower and shorter than open techniques. In this present study, estimated blood loss and operation time were higher than reported in previous studies; however, postoperative hospital stay was comparable [8, 15]. These findings could be explained by the fact that most of the procedures were performed by low-volume surgeons who were at the beginning of their learning curve. Additionally, the rates of urinary tract infection and blood transfusion were comparable to what Ng and Chern reported in their study [26]. Experienced surgeons and estimated blood loss are predictors of a successful laparoscopic hysterectomy [27]. In this present study, estimated blood loss was found to be a predictor of intraoperative complications, and this prediction was also reported in previous studies [28, 29]. The authors believe that as most of the procedures were performed by low-volume surgeons and the surgical field might be obscured with blood; these two factors have led to reported complications.

### 4.1. Study Limitation

The possible presence of perspective bias limits this study since several confounding factors may not have been recognized. In addition, most of the procedures were performed by different surgeons with a wide range of experience. Additionally, it is possible that some of the postoperative complications went unnoticed, as some patients might have been evaluated at a different hospital. Finally, being a single-center study is considered a limitation as it could impact the generalizability of the study`s findings over other settings.

## Figures and Tables

**Table 1:** Demographic and baseline characteristics of the cohort study (*n* = 279).

Variable	Range (mean ± SD)	No. (%)
Age (years)	17–85 (53.3 ± 8.8)	
BMI (kg/m^2^)	18–69 (33.7 ± 6.6)	
Parity		11 (3.9)
0		43 (15.4)
1–4–6		91 (32.6)
> 6		134 (48.1)
Previous laparotomy		
No		180 (64.5)
Yes		99 (35.5)
Chronic medical disease^∗^		
None		60 (21.5)
Diabetes mellitus		99 (35.5)
Thyroid disease		41 (14.7)
Hypertension		95 (34.1)
Others		84 (30.1)

*Note:* Not mutually exclusive (i.e., sum exceeds 100%).

Abbreviations: BMI = body mass index; SD = standard deviation.

**Table 2:** Procedure-related characteristics of the cohort study (*n* = 279).

Variable	Range (mean ± SD)	No. (%)
*Date of procedure*		
2017		35 (12.5)
2018		58 (20.8
2019		52 (18.6)
2020		49 (17.6)
2021		85 (30.5)

*Type of anesthesia*		
General		275 (98.5)
Regional		3 (1.1)
Combined		1 (0.4)

*Use of preoperative prophylactic antibiotic*		
No		3 (1.1)
Yes		276 (98.9)

*Use of prophylactic anticoagulants*		
No		6 (2.2)
Yes		273 (97.8)

*Indications*		
Uterine leiomyoma		55 (19.7)
Pelvic organ prolapse		58 (20.8)
Abnormal uterine bleeding		84 (30.1)
Adenomyosis		22 (7.9)
Endometriosis		6 (2.2)
Postmenopausal bleeding		55 (19.7)
Chronic pelvic pain		3 (1.1)
Others		11 (3.9)

*Intraoperative complications*		
None		259 (92.8)
Bowel injury		2 (0.7)
Ureteric injury		2 (0.7)
Vessel injury		1 (0.4)
Blood transfusion		7 (2.5)
Conversion to laparotomy		7 (2.5)
Others		1 (0.4)

*Postoperative complications*		
None		260 (93.1)
Fever		3 (1.1)
Bowel obstructions		1 (0.4)
Venous thrombosis		1 (0.4)
Urinary tract infection		3 (1.1)
Others		11 (3.9)

Duration of the procedure (hours)	1–7 (2.9 ± 1.0)	

Estimated blood loss (mL)	70–900 (263.0 ± 173.1)	

Postoperative hospital stays (days)	1–25 (2.2 ± 1.8)	

*Note:* Not mutually exclusive (i.e., sum exceeds 100%).

Abbreviation: SD = standard deviation.

**Table 3:** Binary logistic regression for the factors associated with intraoperative complications.

	B	SE	Wald	*p* value	Adjusted odds ratio	95% CI
Age of the patient	−0.036	0.038	0.871	0.351	0.965	0.90–1.04
BMI	0.044	0.034	1.643	0.200	1.045	0.98–1.12
Previous laparotomy	−0.468	0.521	0.807	0.369	0.626	0.23–1.47
Chronic medical disease	0.491	0.690	0.507	0.477	1.635	0.42–6.32
Estimated blood loss	0.005	0.001	15.237	< 0.001	1.005	1.001–1.01
Constant	−3.849	2.162	3.169	0.075	0.021	

*Note:* Variable(s) entered on Step 1: age of the patient, BMI, previous laparotomy, chronic medical disease, and estimated blood loss (mL). B: slope, SE: standard error, df: degree, and f: freedom.

**Table 4:** Binary logistic regression for the factors associated with postoperative complications.

	B	SE	Wald	*p* value	Odds ratio	95% CI
Age of the patient	−0.002	0.033	0.005	0.946	0.998	0.94–1.06
BMI	0.005	0.035	0.020	0.887	1.005	0.94–1.08
Previous laparotomy	−0.322	0.515	0.391	0.532	0.725	0.26–1.99
Chronic medical disease	0.019	0.616	0.001	0.976	1.029	0.30–3.41
Estimated blood loss	0.003	0.001	8.158	0.004	1.003	1.001–1.006
Constant	−3.663	2.019	3.292	0.070	0.070	

*Note:* Variable(s) entered on Step 1: age of the patient, BMI, previous laparotomy, chronic medical disease, and estimated blood loss (mL). B: slope, SE: standard error, df: degree, and f: freedom.

## Data Availability

The data that support the findings of this study are available from the corresponding author upon reasonable request.
